# Effects of royal jelly on ovary cancer cells proliferation and apoptosis

**DOI:** 10.1007/s12032-025-02638-z

**Published:** 2025-03-04

**Authors:** Ender Deniz Asmaz, Sabire Güler, Berrin Zık

**Affiliations:** 1 Department of Histology and Embryology, Faculty of Medicine Ankara Medipol University Ankara, Ankara, Turkey; 2 Department of Histology Embryology, Faculty of Veterinary Medicine, Uludag University, Bursa, Turkey

**Keywords:** Apoptosis, Ovarian cancer, Proliferation, Royal jelly

## Abstract

The aim of the present study is to investigate the proliferative or apoptotic effects of different doses and durations of Royal jelly (RJ) on serous type epithelial ovarian cancer, which is the most common epithelial ovarian cancer. For this purpose, cells of the Skov-3 human ovarian adenocarcinoma cell line were grown in McCoy medium and seeded in 6-well plates. RJ was prepared as a stock solution (1000 mg RJ/10 ml dH_2_O) and 1, 5, 10, 20, and 50 mg/ml RJ doses from the prepared stock solution were added to the medium for 24, 48, and 72 h incubated. After the treatment of RJ, the cell viability test (Tripan Blue), Ki-67 to determine the proliferative effect, cleaved-Caspase-3 and cleaved PARP expressions to determine its apoptotic effect were examined by immunocytochemical and immunofluorescence methods. In addition, findings were supported by the TUNEL method. As a result of the experiments, it was determined that 1 mg/ml and 24 h treatment of RJ did not affect cell proliferation and apoptosis, but generally, 50 mg/ml of RJ for 72 h inhibited proliferation in cancer cells and induced apoptosis. The use of royal jelly both monotherapeutically and in combination as an alternative treatment for ovarian cancer may provide the basis for new experimental protocols.

## Introduction

Cancer ranks second after cardiovascular diseases as the cause of death threatening human health [1]. Ovarian cancer, one of these cancer types, ranks fourth among the most common cancer types in women and is of clinical importance due to its high mortality rates [2, 3]. Ovarian cancers are classified into 3 groups: epithelial ovarian cancers (EOC), gonadal stromal tumors and germ cell tumors. Epithelial ovarian cancer is approximately 90% of all ovarian malignant diseases and is one of the leading causes of death in gynecological cancers [4]. EOCs, according to the cell types in the reproductive system classified into five groups; Serous (incidence: 7/10), mucinous (incidence: 1/10), endometrioid (incidence: 1/20), clear cell (incidence: 3/100), and transitional cell (incidence: 1/10). Treatment is based on a combination of surgery and chemotherapy. Most advanced ovarian cancer patients develop resistance to chemotherapy drugs used after surgery and tumor recurrence occurs over time [3].

Invasion of cancerous cells to surrounding tissues during treatment, development of resistance to chemotherapeutic drugs, and tumor recurrence over time lead researchers to seek the use of a natural compound with strong antioxidant, antitumor, or anticarcinogenic properties that can be protective before or alongside chemotherapeutic agents. *Aronia melanocarpa* (black currant cherry) fruit, which has antioxidant and anthocyanin properties, causes antiproliferative effects in colon cancer [5, 6], *Hypericum olympicum* (Uludağ cantoron) and *Hypericum adenotrichum* (Cranberry grass) strains are reported to show cytotoxic, genotoxic, and apoptotic properties in human lung cancer cell lines (A549 and PC3) [7].

It is reported that Royal jelly (RJ) has many biological activities such as antitumor [8, 9], antiviral, antibacterial, anticarcinogenic [10–13], antiallergic [14], immunomodulatory [15, 16], estrogenic [17, 18] and antidiabetic [19]. RJ is the best food of queen honey bee (*Apis mellifera*) larvae and is secreted from the hypopharyngeal and mandibular glands of worker honey bees between the sixth and twelfth days of life [20]. RJ is a viscous, bone-colored substance with a distinctive smell and burning taste. Chemically, RJ consists of 50–60% water, 18% proteins (Glutamic acid, Aspartic acid, Serine, Leucine, Phenylalanine, Tyrosine, Threonine, Lysine, Valine, Glycine, Methionine, Isoleucine, Proline, Cystine), 15% carbohydrates (Fructose, Glucose, Sucrose), 3–6% lipids and other fatty acids, 1.5% minerals (Potassium, Calcium, Magnesium, Zinc, Iron, Copper, Sodium, Selenium), and many vitamins (Thiamine, Riboflavin, Pantothenic acid, Pyridoxine, Niacin, Folic acid, Inositol, Biotin) [21]. In addition, RJ contains 10-hydroxy-2-decenoic acid (10-HDA), a major and unique fatty acid, which is not found in other bee products (propolis, pollen). 10-HDA has many potential pharmacological effects such as antitumor and angiogenesis inhibition [22], and immunomodulatory activities [23]. Researchers observed that the combination of RJ and interferon-alpha (HuIFN-αN3) showed an antiproliferative effect in human colorectal adenocarcinoma cells (Caco-2) [24]. There are many studies on MCF-7 cancer cells with RJ in combination with other chemotherapeutic drugs. It has been reported that combinations with RJ and Bisphenol A [9], as well as RJ and Tamoxifen [25] suppress the proliferation of MCF-7 cancer cells. In another study with the glioblastoma multiform (U87MG) cell line, it was shown that the combination of 30 mg/mL RJ with 20 mM Temozolomide (TMZ) inhibited *DNA* synthesis in cancer cells [26]. In an in vivo study, it was found that RJ administered orally to experimental animals with fibrocarcinoma at doses of 100, 200, and 300 mg/kg reduced tumour sizes in the experimental group compared to the control group [27]. It also has a therapeutic effect of RJ on leukemia cells [28]. Additionally, a presented study reported that RJ administered to mice with Ehrlich-Lettre acid carcinoma at doses of 500, 1000, and 1500 mg/kg for 33 days both had antitumor effects and modulated the body immune system [8].

In addition to the positive effects of RJ on cancer cells, it also positively affects fertility in both males and females by increasing the quality of oocyte and sperm in the reproductive system [29]. In a study by Kridli et al. (2003) in sheep, it was stated that RJ plays an important role in female fertility with its estrogenic effect [30], while in another study, it was stated that RJ induced spermatogenesis by increasing sperm volume, spermatozoon mobility, and concentration [31]. RJ affects ovarian hormone levels in immature female rats, increasing progesterone/estrogen levels and inducing folliculogenesis [18]. However, RJ has positive effects on oocyte maturation [17].

In recent years, RJ, which has been used both in the reproductive system and in the treatment of different types of cancer, there is no study has been found on its effect on ovarian cancer cells. Therefore, the aim of the presented study is to determine for the first time the time- and dose-dependent effects of RJ on Skov-3, one of the ovarian cancer cell lines, by evaluating both in terms of proliferation and apoptosis.

## Material and method

### Experimental procedure

#### Cell culture

The RJ used in our study was obtained purely from the same hive from Bursa Uludağ University Beekeeping Development-Application and Research Center. Cells in the Skov-3 human ovarian adenocarcinoma cell line were incubated in McCoy [32] medium containing 10% fetal bovine serum (FBS) and 0.1% penicillin–streptomycin in an incubator at 37 °C and 5% CO2 until they proliferated in sufficient numbers. When the cells proliferate sufficiently, they are seeded on 6-well plates and when it reaches 30% prevalence, 1000 mg of RJ is dissolved in 10 ml of distilled water (stock solution) [24] at appropriate concentrations (1, 5, 10, 20, 50 mg/ml) was added to the medium. Cells were incubated in media containing RJ for 24, 48, and 72 h.

### Trypan blue exclusion assay

Following treatment cells were collected. Samples were centrifuged at 1300 rpm for 5 min. Then, pellets were resuspended in medium, and trypan blue (Sigma T6146) was added to the cell suspension in a 1:1 ratio. After, cells were counted using a Thoma slide under the inverted microscope at 10 × magnification. Cells stained blue were counted as dead, and results are expressed as a percentage of total cells. Cell viability was reported as an average of two independent experiments, each condition in duplicates.

### Immunostaining

Cells were grown on cover slips in 24-well plates. After 24, 48, and 72 h, cells on the cover slips were washed three times in phosphate-buffered saline (PBS). The cells then were fixed in 10% Neutral buffer formaldehyde at room temperature for 15 min and permeabilize with 0.1% Triton X-100 for 10 min. For immunocytochemistry (ICC), the cells were blocked (Vector Lab.; MP7401) for 20 min and followed by incubation with proliferation markers Ki-67 (Lab Vision SP6 (dilution 1/500)), cleaved caspase-3 (Cell Signaling ASP175-9661 (dilution 1/200) and cleaved Poly (ADP-ribose) polymerase (PARP) (Santa-Cruz Biotechnology, sc-23461-R (dilution 1/300)) antibody at 4 °C overnight, then incubation with secondary antibody (Vector Lab.; MP7401) for 30 min. Cells were then treated with 3,3′- Diaminobenzidine (DAB) (Invitrogen:00-2020) for 5 min and counterstained with Harris Hematoxilen for 2 min, and the images were observed under the Nikon Eclipse 80i microscope.

For immunofluorescence (IF), the cells were blocked with 5% bovine serum albumin (BSA) (in PBS) for 1 h and followed by incubation with Ki-67 (dilution 1:500), cleaved caspase-3 (dilution 1:200) antibody at 4 °C overnight, then incubation with secondary antibody (dilution 1:2000) (Alexa Fluor 488 Conjugate 4412S) for 1 h in a dark room. Then, cover slips mounted and visualized under the fluorescence attached Nikon Eclipse 80i microscope.

### TUNEL assay

To confirm the expression of cleaved caspase-3 and cleaved PARP in cells, Terminal Deoxynucleotidyl Transferase Mediated dUTP Nick End Labeling (TUNEL) method was applied according to the protocol in TUNEL Apoptosis Kit (Roche Diagnostics GmbH, Mannheim, Germany). The cells were seeded in cover slides (4 × 10^4^ cells per slide) and then treated with RJ at different concentrations (1, 5, 10, 20, and 50 mg/ml). Following this, the cells were washed in PBS and prepared 10% formaldehyde was added for cell fixation for 20 min. The cells were rewashed in PBS, before being permeabilized in permeabilization solution (0.1% Triton X-100) for 5 min on ice. The permeabilized cells were incubated in the dark at 37 °C for 1 h with the TUNEL reaction mixture containing terminal deoxynucleotidyl transferase (TdT) plus dUTP label. After labeling, cells were washed with PBS and incubated with TUNEL-POD solution for 30 min at 37 °C that to view the cells under light microscopy. Cells washed in PBS were treated with DAB (3.3 diaminobenzidine) for 10 min for peroxidase coloring reaction. Cells were then counterstained with Harris hematoxylin rinsed in distilled water, and examined under microscope (Nikon Eclipse 80i; Tokyo, Japan). Negative control sections were incubated only in TUNEL label solution without adding enzyme (terminal deoxynucleotidyl transferase (TdT)). Positive control sections received the same treatment but were pre-treated with DNase I (Roche, Indianapolis, USA) for 30 min at 37 °C.

### Quantitative evaluation of immunostaining and TUNEL

Cells treated with RJ at the end of each dose and time were evaluated by two independent observers after ICC, IF, and TUNEL staining.

-Calculation of the proliferation index; Ki-67 positive and negative cells were counted in 5 randomly selected areas on at least 3 coverslips; The number of Ki-67 positive cells/total cell count was calculated as × 100.

-Calculation of the apoptotic index; TUNEL, cleaved caspase-3 and cleaved PARP positive and negative cells were counted on at least 3 coverslips in 5 randomly selected areas; The number of apoptotic cells/total cell number was calculated as × 100 [33–35].

### Statistical analysis

Statistical analyzes of the data obtained from the study were performed using the SPSS 23.0 (Statistical Package for Social Sciences) program. Data were examined for normality distribution and variance homogeneity assumptions (Shapiro–wilk test). Statistical significance between the groups was analyzed by one-way ANOVA test followed by Dunn’s post hoc test. The symbols *p* ≤ 0.05 were used to indicate the level of confidence.

## Results

**Fig. 1:**
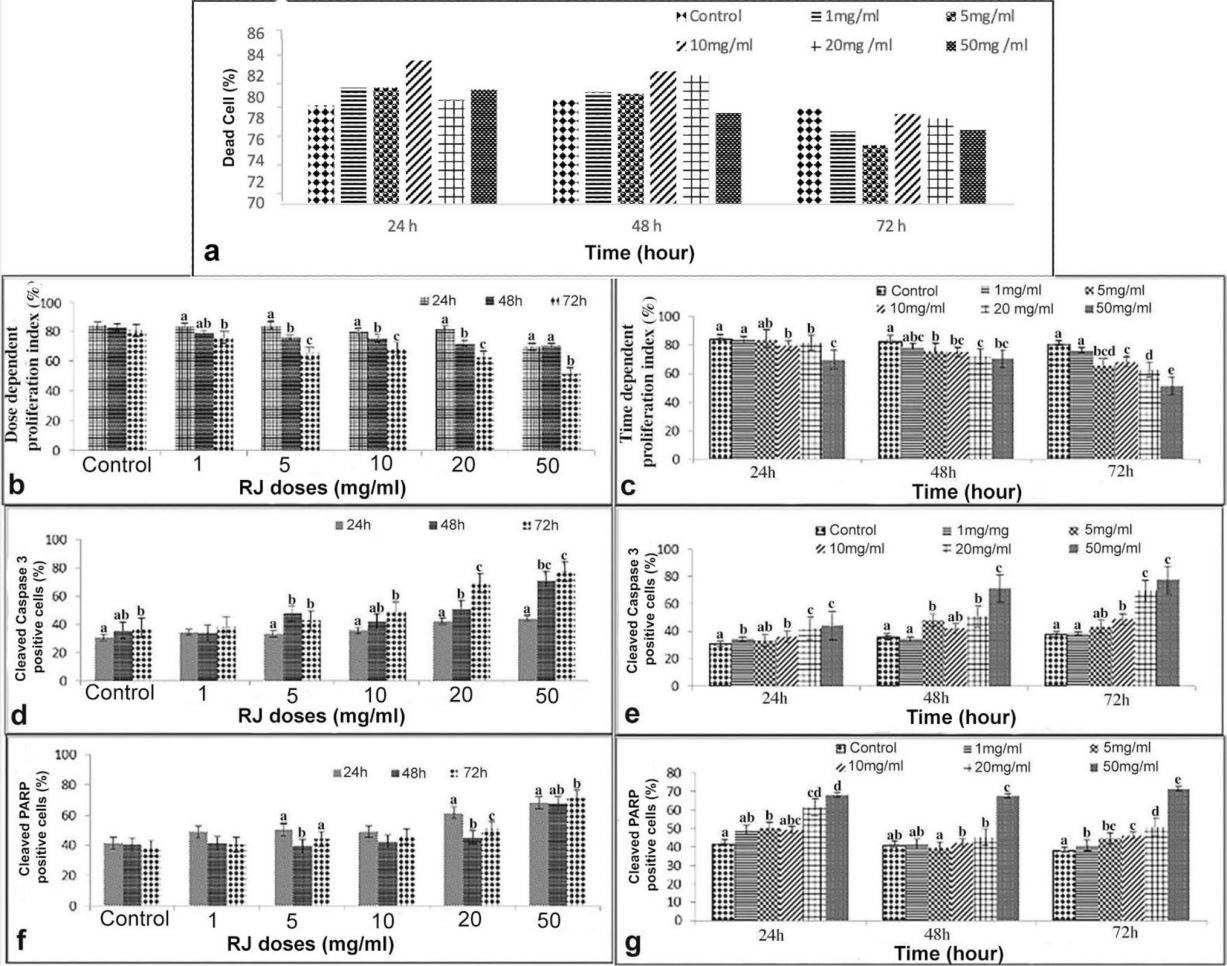
Effect of RJ on Skov 3; **a** Time and dose-dependent cell viability values of RJ (%); **b** Dose-dependent Ki-67 immunoreaction at different times; **c** Time-dependent Ki-67 immunoreaction of different doses; **d** Dose-dependent cleaved caspase-3 immunoreaction at different times; **e** Time-dependent cleaved caspase-3 immunoreaction of different doses; **f** Dose-dependent cleaved PARP immunoreaction at different times; **g** Time-dependent cleaved PARP immunoreaction of different doses. Different letters across doses are statistically significant (*p* ≤ 0.05), *h* hour

The SKOV-3 ovarian cancer cell line was treated at different concentrations of RJ (1, 5, 10, 20, and 50 mg/ml) and different time (24, 48, 72 h). At the end of the period, viability tests were performed with trypan blue. No statistical significance was observed time and concentration-dependent in all groups (*p* ≥ 0.05) (Fig. 1a).

### ICC test results

#### Cell proliferation

**Fig. 2:**
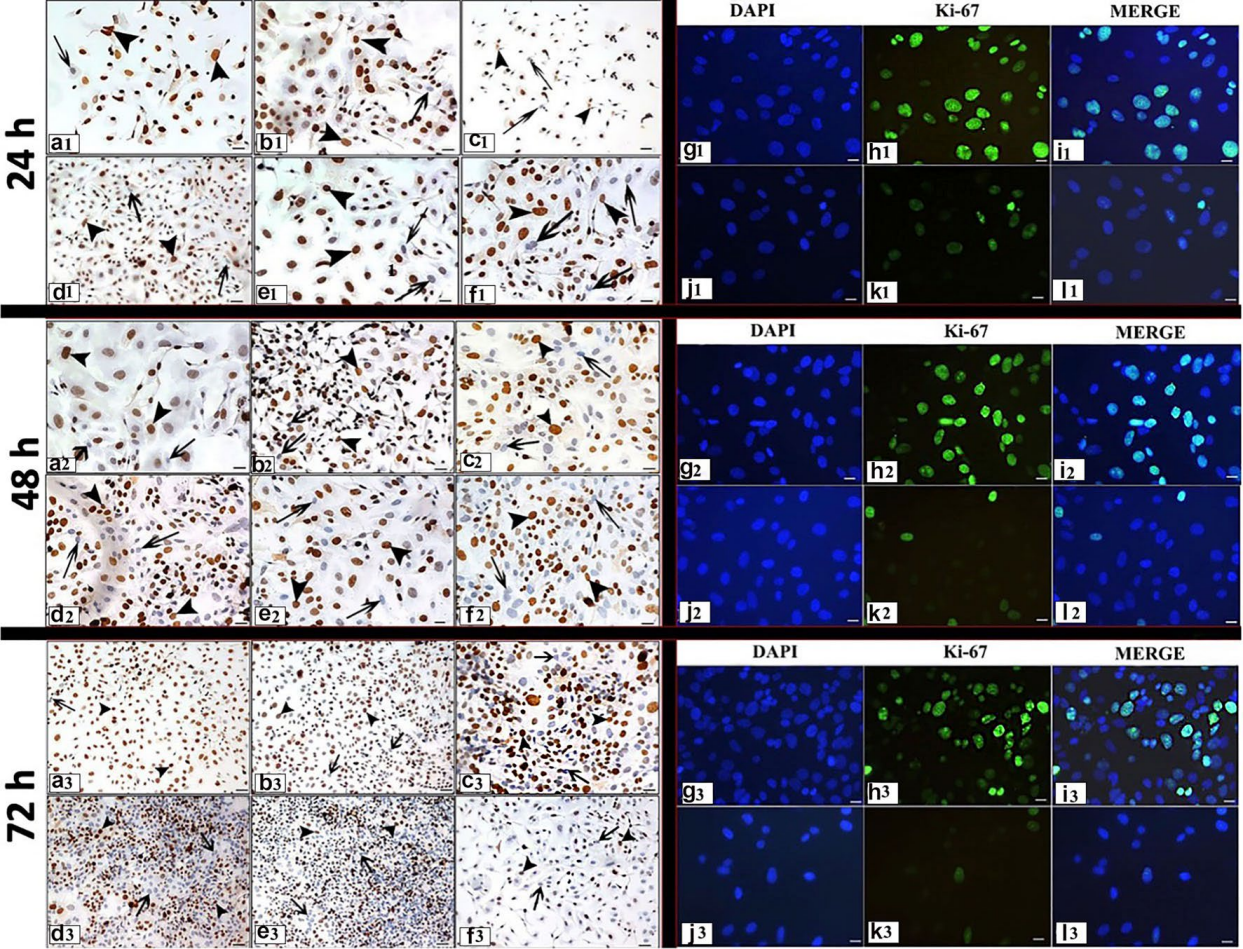
Ki-67 expression following 24, 48, 72 h RJ treatment. **a1, a2, a3:** Control group, **b1, b2, b3:** 1 mg/ml dose RJ, **c1, c2, c3:** 5 mg/ml dose RJ, **d1, d2, d3:** 10 mg/ml dose RJ, **e1, e2, e3:** 20 mg/ml dose RJ, **f1, f2, f3:** 50 mg/ml dose RJ. **g1, g2, g3, h1 h2 h3, i1, i2, i3:** Control group, **j1, j2, j3, k1, k2, k3, l1, l2, l3:** 50 mg/ml dose RJ, **g**–**j:** Cell nuclei stained with DAPI, **h1, h2, h3, k1, k2, k3:** Ki-67 protein expression, **i1, i2, i3, l1, l2, l3:** Merge. arrow: Ki-67 negative reaction, arrowhead: Ki-67 positive reaction. Immunocytochemical staining/ Immunofluorescence staining Bar 50 µm

The dose- and time-dependent proliferation index (Ki-67) of RJ in Skov-3 cancer cell line was evaluated by ICC and IF staining method. Ki-67 expression was observed in the nuclei of the cells (Fig. 2). At the end of the experiment, the proliferative index differences between the groups are presented depending on both doses and times (Fig. 1b, c).

The treatment of the only 1 mg/ml dose RJ for 72 h caused a decrease in cell proliferation compared to the administration time of 24 h of same dose (Figs. 1b, 2). However, the other doses of RJ suppressed cell proliferation in a time-dependent manner (*p* ≤ 0.05). Treatment with 50 mg/ml RJ for 72 h showed the lowest proliferative effect in all doses and times (*p* ≤ 0.05) (Figs. 1b, c, 2).

After 24 h treatment, the proliferation rate in cells treated with higher doses of RJ was markedly reduced (*p* ≤ 0.05). It was observed that cell proliferation was suppressed with increasing dose (Figs. 1c, 2).

After 48 h, while a statistical significance was determined between the control group and other doses RJ groups except for lowest dose RJ (*p* ≤ 0.05), no statistical significance was observed between the doses of 10, 20, and 50 mg/ml RJ (*p* ≥ 0.05). The lowest proliferation level was determined numerically at the high dose of RJ (Figs. 1b, c, 2).

After 72 h, the proliferation rate of the cells treated with 50 mg/ml RJ was significantly lower than the control and other RJ group’s (*p* ≤ 0.05) (Figs. 1b, c, 2).

Generally, it was observed that cell proliferation was suppressed with increasing dose and duration of RJ.

#### Cell apoptosis

**Fig. 3:**
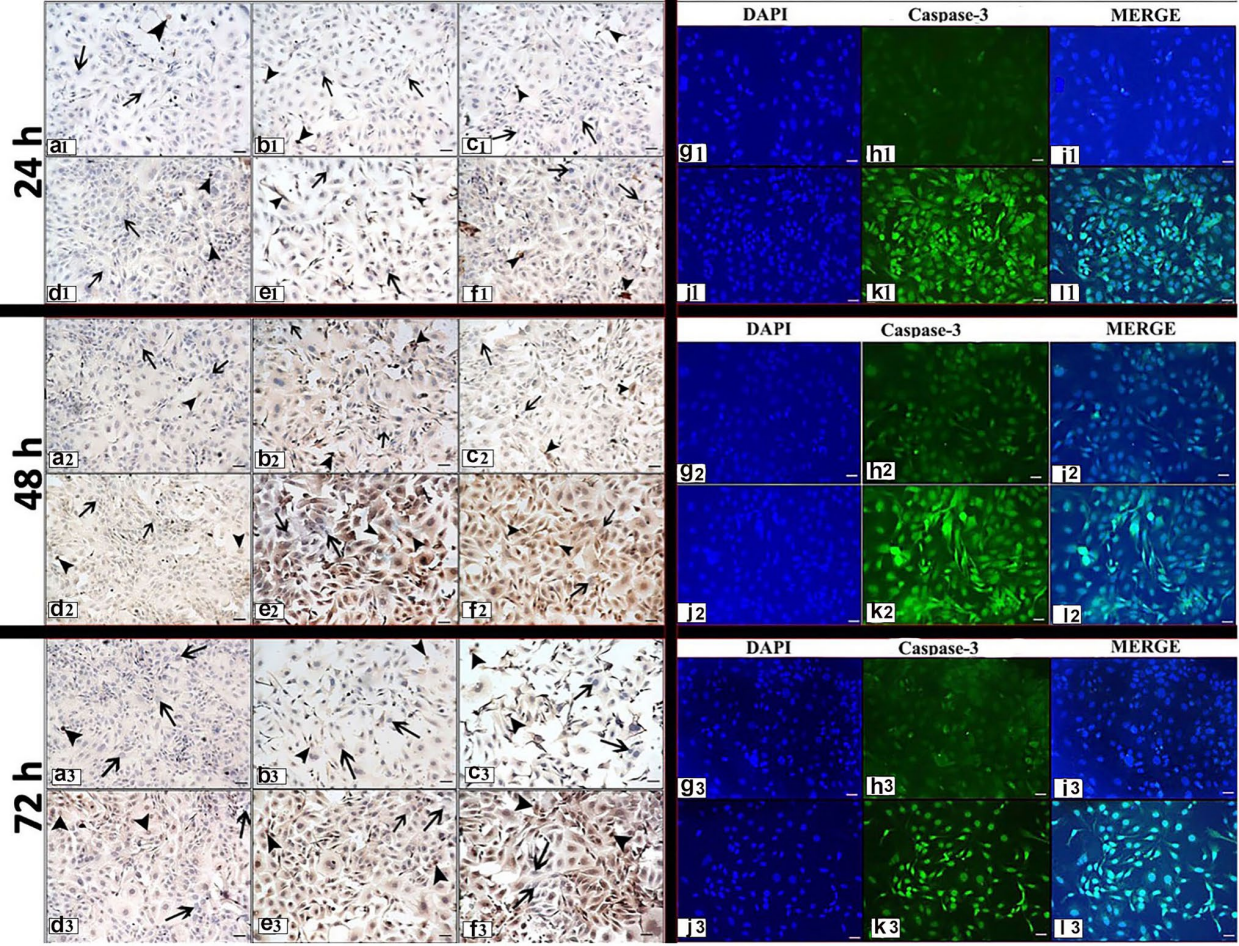
Cleaved caspase-3 expression following 24, 48, 72 h RJ treatment**. a1, a2, a3:** Control group, **b1, b2, b3:** 1 mg/ml dose RJ, **c1, c2, c3:** 5 mg/ml dose RJ, **d1, d2, d3:** 10 mg/ml dose RJ, **e1, e2, e3:** 20 mg/ml dose RJ, **f1, f2, f3:** 50 mg/ml dose RJ. **g1, g2, g3, h1 h2 h3, i1, i2, i3:** Control group, **j1, j2, j3, k1, k2, k3, l1, l2, l3:** 50 mg/ml dose RJ, **g**–**j:** Cell nuclei stained with DAPI, **h1, h2, h3, k1, k2, k3:** Cleaved caspase-3 protein expression, **i1, i2, i3, l1, l2, l3:** Merge. arrow: Cleaved caspase-3 negative reaction, arrowhead: Cleaved caspase-3 positive reaction. Immunocytochemical staining/ Immunofluorescence staining Bar 50 µm

**Fig. 4:**
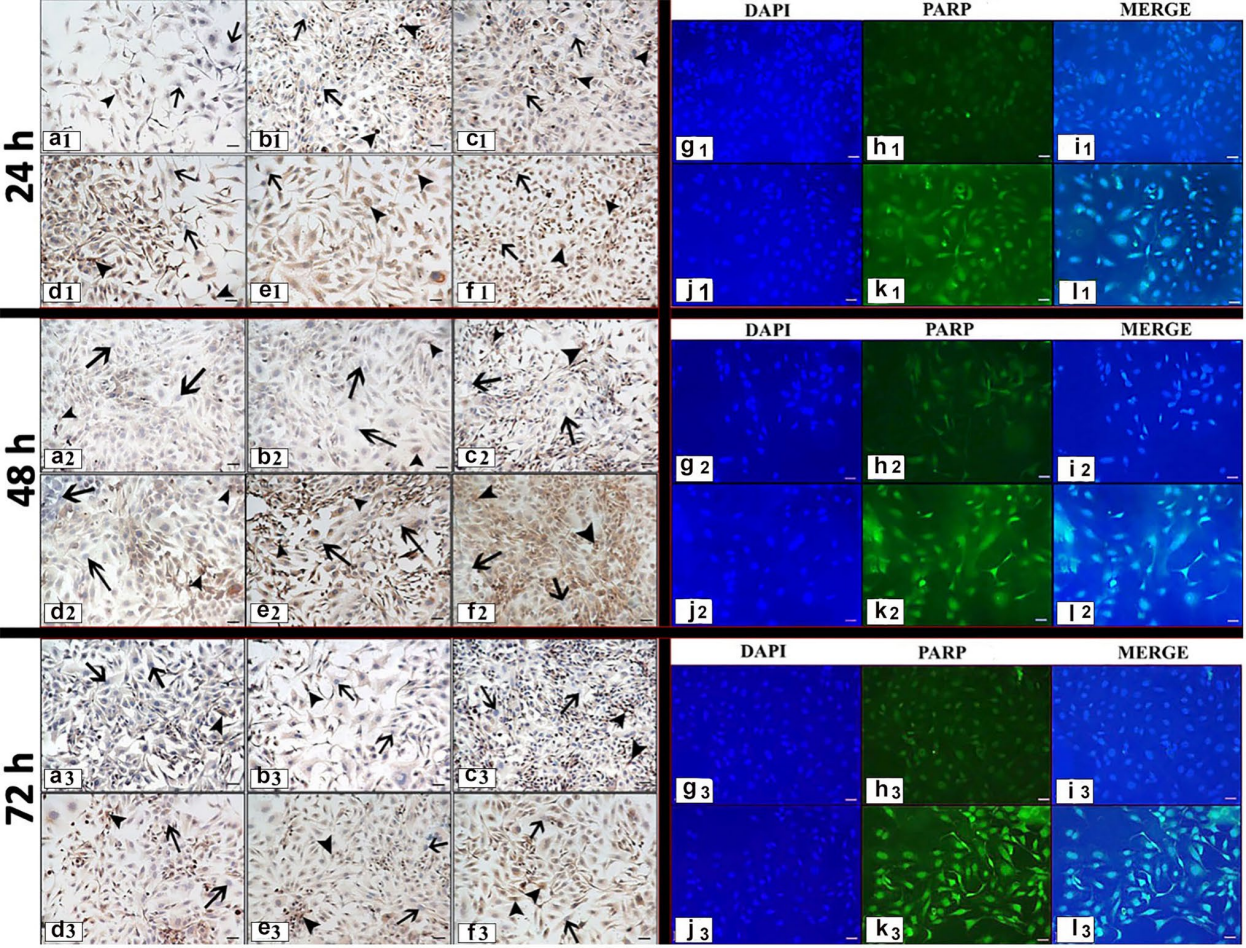
Cleaved PARP expression following 24,48,72 h RJ treatment**. a1, a2, a3:** Control group, **b1, b2, b3:** 1 mg/ml dose RJ, **c1, c2, c3:** 5 mg/ml dose RJ, **d1, d2, d3:** 10 mg/ml dose RJ, **e1, e2, e3:** 20 mg/ml dose RJ, **f1, f2, f3:** 50 mg/ml dose RJ. **g1, g2, g3, h1, h2, h3, i1, i2, i3:** Control group, **j1, j2, j3, k1, k2, k3, l1, l2, l3:** 50 mg/ml dose RJ, **g**–**j:** Cell nuclei stained with DAPI, **h1, h2, h3, k1, k2, k3:** Cleaved PARP protein expression, **i1, i2, i3, l1, l2, l3:** Merge. arrow: Cleaved PARP negative reaction, arrowhead: Cleaved PARP positive reaction. Immunocytochemical staining/ Immunofluorescence staining Bar 50 µm

**Fig. 5:**
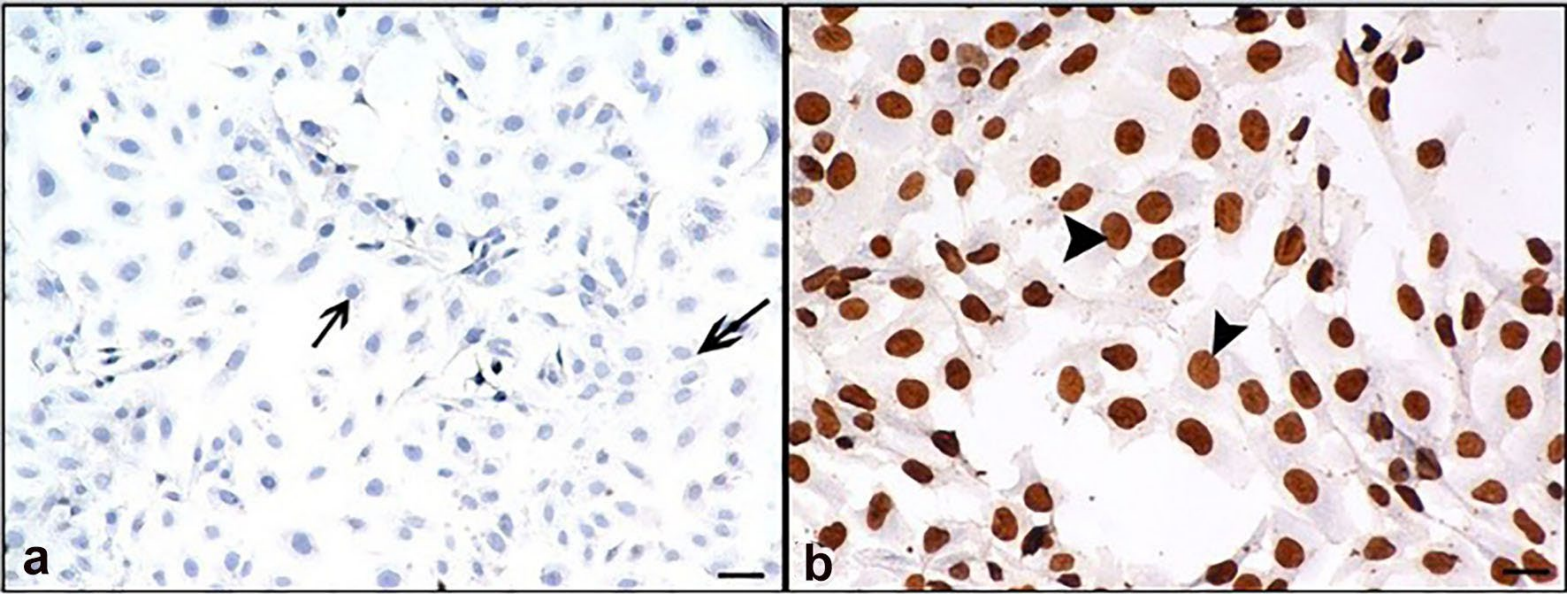
Result of 24-h RJ treatment. **a** TUNEL negative control group, where only label solution was applied without adding enzyme (TdT); **b** TUNEL positive control group, where DNase was applied. arrow: TUNEL negative reaction, arrowhead: TUNEL positive reaction. Bar 50 µm

**Fig. 6:**
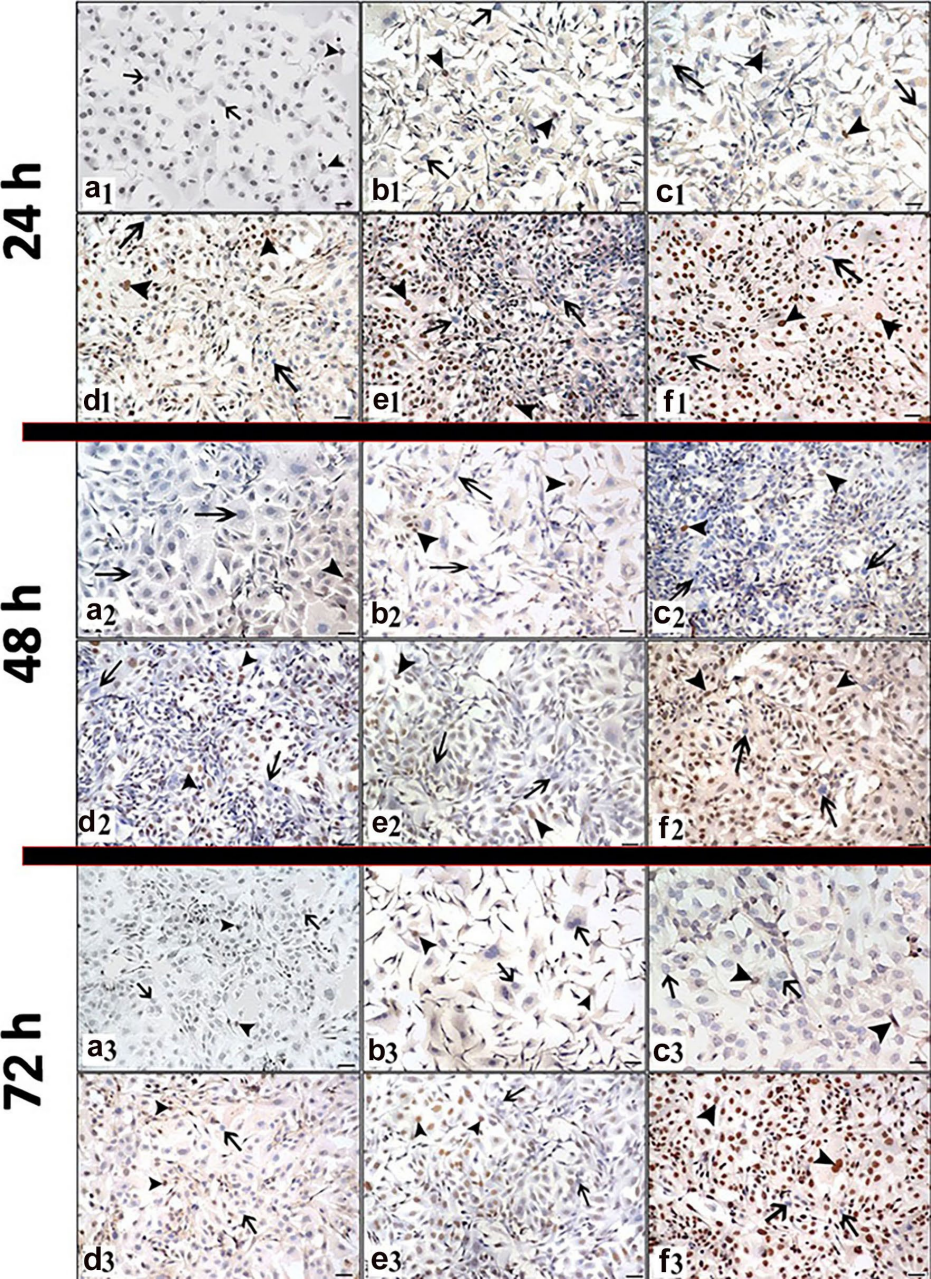
TUNEL expression following 24, 48,72 h RJ treatment. **a1, a2, a3:** Control group, **b1, b2, b3:** 1 mg/ml dose RJ, **c1, c2, c3:** 5 mg/ml dose RJ, **d1, d2, d3:** 10 mg/ml dose RJ, **e1, e2, e3:** 20 mg/ml dose RJ, **f1, f2, f3:** 50 mg/ml dose RJ. arrow: Tunel negative reaction, arrowhead: Tunel positive reaction. Immunocytochemical staining, Bar 50 µm

The apoptotic effects of different concentrations RJ on Skov-3 ovarian cancer line, the expressions of cleaved caspase-3 and cleaved PARP were determined by ICC and IF staining (Figs. 3, 4). Additionally, the TUNEL method was applied to determine the apoptotic effects of RJ (Figs. 5, 6).

### Cleaved caspase-3

Cleaved caspase-3 immunoreaction was observed both in the cell nucleus and cytoplasm by immunocytochemical and immunofluorescent staining (Fig. 3). At the end of the experiment, differences between groups and apoptotic index are presented both times and doses dependent (Fig. 1d, e). When the apoptotic effect of dose-dependent RJ treatment on Skov-3 ovarian cancer cells was examined, no statistical difference was observed time-dependent manner in 1 mg/ml RJ dose group (*p* ≥ 0.05). However, it was observed that cleaved caspase-3 expression was high in other RJ doses (except 1 mg/ml), especially in the 48 and 72 h treatment (Figs. 1d, e, 3).

After 24 h treatment, a higher cleaved caspase-3 expression was determined at doses of 20 and 50 mg/ml RJ than at all other doses (*p* ≤ 0.05), (Figs. 1e, 3).

After 48 h treatment, while no significant difference was observed between the control group and 1 and 10 mg/ml RJ doses (*p* ≥ 0.05), statistical significance was determined between the control group and the other RJ groups (*p* ≤ 0.05). The highest cleaved caspase-3 expression was determined in treatment of 50 mg/ml RJ (*p* ≤ 0.05) (Figs. 1e, 3).

After 72 h treatment, While no significant difference was observed between 20 and 50 mg/ml RJ doses (*p* ≥ 0.05), statistical difference was observed between these two doses and the all other groups (control group, 1, 5, 10 mg/ml RJ) (*p* ≤ 0.05) (Figs. 1e, 3).

### Cleaved PARP

Cleaved PARP which is involved in a different step of the apoptosis pathway, immunoreaction was observed both in the cell nucleus and cytoplasm in immunocytochemical and immunofluorescent staining (Fig. 4).

At the end of the experiment, differences between groups and apoptotic index are presented depending on both doses and times (Fig. 1f, g). No statistical significance was observed in a time-dependent manner in the control group and treatment with 1 and 10 mg/ml RJ doses (*p* ≥ 0.05). For the 5 mg/ml RJ dose, a statistical difference was observed between the 24 h and 48 h treatment periods (*p* ≤ 0.05) also 20 mg/ml RJ dose showed statistical significance between all treatment periods (*p* ≤ 0.05).

After 24 h treatment, statistical significance was observed between the control group and 5, 20, and 50 mg/ml RJ doses (*p* ≤ 0.05). While no significant difference was observed between the 20 and 50 mg/ml RJ doses (*p* ≥ 0.05), statistical significance was determined between the 50 mg/ml RJ dose and other groups except 20 mg/ml RJ dose (*p* ≤ 0.05) (Figs. 1g, 4).

After 48 h treatment, a significant difference was determined between 50 mg/ml RJ and all groups (*p* ≤ 0.05) (Figs. 1g, 4).

Similar to the 48 h RJ treatment, statistical significance was observed between the highest dose and other groups after 72 h treatment (*p* ≤ 0.05) (Figs. 1g, 4).

### TUNEL results

TUNEL negative and positive controls are presented in Fig. 5a, b, respectively. TUNEL positive reaction was observed in cell nuclei (Fig. 5b).

After 24 h and 72 h treatment, TUNEL positive cell density was less dense in treatment the low dose RJ (1and 5 mg/ml). However, as a result of 48 h treatment, TUNEL positive cell density was observed to be higher in the 20 and 50 mg/ml RJ doses compared to the control group and 1, 5, and 10 mg/ml RJ groups (Fig. 6). The most intense tunnel positive cells were observed in the 50 mg/ml RJ dose treatment for all times.

## Discussion

Chemotherapy is widely used for cancer treatment, but the side effects of chemotherapy agents, damage to normal cells and resistance of tumor cells to these agents are the biggest limitations of this treatment method. Therefore, there is a need for supporting natural compounds that may be more effective to overcome these limitations.

It is reported that RJ has many biological activities such as antitumor, antiviral, antibacterial, antiallergic, anticancerogenic, immunomodulatory, estrogenic and antidiabetic [9, 11, 12, 15, 18, 19, 36]. Although the doses and treatment periods used in in vivo/in vitro environments are different, many positive results have been revealed in studies with RJ [12, 18, 36]. However, in studies on cancer, no studies on ovarian cancer were found. For this reason, in the presented study, the proliferative and apoptotic effects of RJ on the serous type epithelial ovarian cancer cell line (Skov-3), the most common epithelial ovarian cancer, were investigated.

When the antiproliferative effect of RJ in different types of cancer is examined, it is reported that RJ has a significant antiproliferative activity in slow-growing cancers, but does not show the same effect in fast-growing cancer types [37]. Nakaya et al. (2007) stated that RJ inhibited the growth-promoting effect of Bisphenol A (BPA), an environmental estrogen, on MCF7 cells, and that RJ disrupted estrogen-induced cell proliferation signals [9].

It has been reported that GE132 + natural combination (Reishi mushroom, RJ, Resveratrol, Lycopene and Sulforaphane containing) showed antiproliferative effects on SW480 (colon cancer cells) and EAhy 926 (normal human endothelial cancer cell line) cell lines at a dose of 750 µg/ml [38]. Although the dose of GE132 + natural combination was lower than the doses used in our study, the antiproliferative effect it showed was due to the RJ combination. In our study, while the antiproliferative effect of RJ was not observed at the lowest dose and duration, antiproliferative effects of RJ on Skov-3 were determined with the increase in duration and dose.

It has been reported that the treatment of 1.5 mM and 5 mM doses of 10HDA, a unique protein compound contained in RJ, to B16F10 melanoma cancer cells for 24 h creates a cytotoxic effect on the cells, but at the same time 0.1, 0.5, and 1 mM 10-HDA concentrations are not cytotoxic in cancer cells [39]. However, since the doses used in our study were lower than the doses used by Peng et al. (2017) a similar result was not found in the cell viability test.

It has been reported that the combination of RJ (0.1 g/ml) with human interferon-alpha (HuIFN-aN3) (1000 I.U. mL-1) at a ratio of 2:1 has a stronger antiproliferative effect on human colorectal adenocarcinoma cells compared to the monotherapeutic treatment of RJ [24]. This study, which supports the antiproliferative efficacy of the highest dose (50 mg/ml) used in our study, suggests that testing RJ with combined drug treatments may produce more effective results.

Apoptosis is one of the main forms of cellular death associated with characteristic morphological changes including chromatin condensation, DNA fragmentation and formation of apoptotic bodies [40]. Cysteine proteases called caspase enzymes (especially caspase 3, 8, and 9) and Bcl-2 family, which contribute to the apoptotic mechanism, play an active role in this process. Caspase-3, the most important member of the caspase family, is responsible for many biochemical mechanisms of apoptosis, leading to cleavage of nuclear and cytosolic substrates, chromatin condensation, DNA fragmentation, and apoptotic bodies [41]. Bee products have been reported to induce in vitro cellular apoptosis in numerous cancer cell lines, including prostate, lung and liver cancers [42]. These biologically active natural products are also recognized as part of an innovative treatment for various types of cancer, including breast and colon cancers [36]. It has been proven in many different studies that RJ has a potential antitumor activity in mice [43], induces apoptotic and antiproliferative pathways in tumor cells, and therefore has an anticarcinogenic activity [44, 45]. In the present study, the expressions of cleaved caspase-3 and cleaved PARP proteins, which are involved in different pathways of apoptosis, were investigated by applying different doses and durations of RJ. The apoptotic effects of RJ on Skov-3 cancer line with the increase in dose concentration and treatment period support the studies conducted with other cancer cells [8, 22].

In a study with the Skov-3 cancer cell line, it was reported that the aqueous extract of the leaves of Solanum nigrum (AE-SN), a natural component such as RJ, induced cleaved caspase-3 [46]. In another study, it was reported that the combination of 30 mg/mL RJ, with 20 mM temozolomide (TMZ) inhibited *DNA* synthesis in cancer cells (glioblastoma multiform (U87MG)), similar to the dose used in our study [26]. In our study, it was determined that similar doses induced apoptosis on the ovarian cancer cell line.

RJ is used in in vivo studies as well as in vitro studies. It is seen that RJ, given orally to experimental animals with fibrocarcinoma at doses of 100, 200, and 300 mg/kg reduces tumor sizes [27]. In addition, RJ administered to mice with Ehrlich-Lettre acid carcinoma at doses of 500, 1000, and 1500 mg/kg for 33 days was seen to have both anti-tumour effects and regulate the body immune system [8]. Although the doses and durations used in in vivo studies differ according to the doses and durations in our study, the results obtained from our study are consistent with both in vivo and in vitro studies above.

## Conclusion

In the presented study, the anticarcinogenic effects of different doses and durations of RJ were evaluated in terms of both cell proliferation and apoptosis, and the anticarcinogenic effect of RJ on Skov-3 cancer cell line was revealed for the first time. In our study, the highest dose and duration of RJ treatment on the cancer cell line suppressed cell proliferation, and therefore induced apoptosis in the cells. In the light of the findings, we think that the use of RJ as an alternative treatment on ovarian cancer, both monotherapeutically and in combination, can form the basis for new experimental protocols in future studies.

## Data Availability

No datasets were generated or analysed during the current study.

## Abbreviations

RJ: Royal jelly
DNA: Deoxyribonucleic acid
CO2: Carbon dioxide
mg: Milligram
ml: Milliliter
kg: Kilogram
°C: Celsius
h: Hour
PBS: Phosphate-buffered saline
DAB: 3,3′- Diaminobenzidine
ICC: Immunocytochemistry
IF: Immunofluorescence
BSA: Bovine serum albümin
PARP: Poly (ADP-ribose) polymerase
TUNEL: Terminal Deoxynucleotidyl Transferase Mediated dUTP Nick End Labeling
TdT: Terminal deoxynucleotidyl transferase
DNase: Deoxyribonuclease

## References

[1] Nagai H, Kim YH. Cancer prevention from the perspective of global cancer burden patterns. J Thorac Dis. 2017;9:448–51. 10.21037/jtd.2017.02.75. 28449441 10.21037/jtd.2017.02.75 PMC5394024

[2] Liu XD, Liu Y, Gong TT, Guo JY, Wang YN, Wang L, Wu QJ, Jiao YS. Prognostic influence of the time interval between surgery and chemotherapy in epithelial ovarian cancer. J Cancer. 2018;9:4172–8. 10.7150/jca.27409. 30519317 10.7150/jca.27409 PMC6277625

[3] Shi C, Wang M. LINC01118 modulates paclitaxel resistance of epithelial ovarian cancer by regulating Mir-134/ABCC1. Med Sci Monit. 2018;24:8831–9. 10.12659/MSM.910932. 30521500 10.12659/MSM.910932 PMC6292151

[4] Choi JH, Wong AS, Huang HF, Leung PC. Gonadotropins and ovarian cancer. Endocr Rev. 2007;2018(28):440–61. 10.1210/er.2006-0036. 10.1210/er.2006-0036 17463396

[5] Shakib Khoob M, Hosseini SM, Kazemi S. In vitro and In vivo antioxidant and anticancer potentials of royal jelly for dimethylhydrazine-Induced colorectal cancer in wistar rats. Oxid Med Cell Longev. 2022;2022:9506026. 10.1155/2022/9506026. 35910834 10.1155/2022/9506026 PMC9334054

[6] Jurikova T, Mlcek J, Skrovankova S. Fruits of black chokeberry *Aronia melanocarpa* in the prevention of chronic diseases. Molecules. 2017;2:944. 10.3390/molecules22060944. 10.3390/molecules22060944 PMC6152740 28590446

[7] Aztopal N, Erkisa M, Celikler S, Ulukaya E, Ari F. Antigrowth and apoptosis inducing effects of Hypericum Olympicum L. and Hypericum Adenotrichum Spach. on lung cancer cells in vitro: Involvement of DNA damage. J Food Biochem. 2016;40:559–66. 10.1111/jfbc.12248.

[8] Bincoletto C, Eberlin S, Figueiredo CA, Luengo MB, Queiroz ML. Effects produced by Royal Jelly on haematopoiesis: relation with host resistance against Ehrlich ascites tumour challenge. Int Immunopharmacol. 2005;5:679–88. 10.1016/j.intimp.2004.11.015. 15710337 10.1016/j.intimp.2004.11.015

[9] Nakaya M, Onda H, Sasaki K, Yukiyoshi A, Tachibana H, Yamada K. Effect of royal jelly on bisphenol A-induced proliferation of human breast cancer cells. Biosci Biotechnol Biochem. 2017;71:253–5. 10.1271/bbb.60453. 10.1271/bbb.60453 17213647

[10] Bagameri L, Baci GM, Dezmirean DS. Royal Jelly as a nutraceutical natural product with a focus on its antibacterial activity. Pharmaceutics. 2022;14:1142. 10.3390/pharmaceutics14061142. 35745715 10.3390/pharmaceutics14061142 PMC9227439

[11] Maželienė Ž, Aleksandravičienė A, Pašvenskaitė M, Viliušienė I, Šakienė D, Dailidaitė E. Antimicrobial activity of royal jelly, honey, and their mixture. Biologija. 2022;68:159–64. 10.6001/biologija.v68i3.4786.

[12] Arzi A, Olapour S, Yaghooti H, Sistani-Karampour N. Effect of Royal Jelly on formalin induced- inflammation in rat hind paw. Jundishapur J Nat Pharm. 2015;10:e22466. 10.17795/jjnpp-22466. 10.17795/jjnpp-22466 PMC4385293 25866724

[13] Yang Y, Chou W, Widowati DA. 10-hydroxy-2-decenoic acid of royal jelly exhibits bactericide and anti-inflammatory activity in human colon cancer cells. BMC Complement Altern Med. 2018;18:202. 10.1186/s12906-018-2267-9. 29970062 10.1186/s12906-018-2267-9 PMC6029378

[14] Okamoto I, Taniguchi Y, Kunikata T, Kohno K, Iwaki K, Ikeda M, Kurimoto M. Major royal jelly protein 3 modulates immune responses in vitro and in vivo. Life Sci. 2003;73:2029–45. 10.1016/s0024-3205(03)00562-9. 12899927 10.1016/s0024-3205(03)00562-9

[15] Wu H, Zhou S, Ning W, Wu X, Xu X, Liu Z, Liu W, Liu K, Shen L, Wang J. Major royal-jelly proteins intake modulates immune functions and gut microbiota in mice. Food Sci Hum Wellness. 2024;13:444–53. 10.26599/FSHW.2022.9250038.

[16] Natarajan O, Angeloni JT, Bilodeau MF, Russi KE, Dong Y, Cao M. The immunomodulatory effects of Royal Jelly on defending against bacterial infections in the Caenorhabditis elegans model. J Med Food. 2021;24:358–69. 10.1089/jmf.2020.0050. 32701017 10.1089/jmf.2020.0050

[17] Shen L Sr, Liu X, Chen Y, Shi F, Lai CQ Sr. Major royal jelly proteins accelerate onset of puberty and promote ovarian follicular development in immature female mice. Curr Dev Nutr. 2020. 10.1093/cdn/nzaa055_030.32072130

[18] Ghanbari E, Khazaei MR, Khazaei M, Nejati V. Royal Jelly promotes ovarian follicles growth and increases steroid hormones in immature rats. Int J Fertil Steril. 2018;11:263–9. 10.22074/ijfs.2018.5156. 29043701 10.22074/ijfs.2018.5156 PMC5641457

[19] Nohair SFA. Antidiabetic efficacy of a honey-royal jelly mixture: Biochemical study in rats. Int J Health Sci (Qassim). 2021;4:4–9. PMC8265305 34285683

[20] Liu Y, Jiang B, Wang K. A review of fermented bee products: Sources, nutritional values, and health benefits. Food Res Int. 2023;174(Pt 1):113506. 10.1016/j.foodres.2023.113506. 37986501 10.1016/j.foodres.2023.113506

[21] Botezan S, Baci GM, Bagameri L, Pașca C, Dezmirean DS. Current status of the bioactive properties of Royal Jelly: a comprehensive review with a focus on its anticancer, anti-Inflammatory, and antioxidant effects. Molecules. 2023;3(28):1510. 10.3390/molecules28031510. 10.3390/molecules28031510 PMC9921556 36771175

[22] Izuta H, Chikaraishi Y, Shimazawa M, Mishima S, Hara H. 10-Hydroxy-2- decenoic Acid, a major fatty acid from Royal Jelly, inhibits VEGF induced angiogenesis in human umbilical vein endothelial cells. Evid Based Complement Alternat Med. 2009;6:489–694. 10.1093/ecam/nem152. 18955252 10.1093/ecam/nem152 PMC2781774

[23] Sugiyama T, Takahashi K, Mori H. Royal jelly acid, 10-hydroxy-trans 2-decenoic acid, as modulator of the innate immune response. Endocr Metab Immune Disord Drug Targets. 2012;12:368–76. 10.2174/187153012803832530. 23061418 10.2174/187153012803832530

[24] Filipič B, Gradišnik L, Rihar K, Šooš E, Pereyra A, Potokar J. The influence of royal jelly and human interferon-alpha (HuIFN-αN3) on proliferation, glutathione level and lipid peroxidation in human colorectal adenocarcinoma cells in vitro. Arh Hig Rada Toksikol. 2015;66:269–74. 10.1515/aiht-2015-66-2632. 26751858 10.1515/aiht-2015-66-2632

[25] Mishima S, Suzuki KM, Isohama Y, Kuratsu N, Araki Y, Inoue M, Miyata T. Royal jelly has estrogenic effects in vitro and in vivo. J Ethnopharmacol. 2005;101:215–20. 10.1016/j.jep.2005.04.012. 15946813 10.1016/j.jep.2005.04.012

[26] Borawska MH, Markiewicz-Żukowska R, Naliwajko SK, Moskwa J, Bartosiuk E, Socha K, Surażyński A, Kochanowicz J, Mariak Z. The interaction of bee products with temozolomide in human diffuse astrocytoma, glioblastoma multiforme and astroglia cell lines. Nutr Cancer. 2014;66:1247–56. 10.1080/01635581.2014.951735. 25256634 10.1080/01635581.2014.951735

[27] Shirzad M, Kordyazdi R, Shahinfard N, Nikokar M. Does Royal jelly affect tumor cells? J HerbMed Pharmacol. 2013;2:45–8.

[28] Yazdanparast S, Bashash D, Nikkhah Bahrami A, Ghorbani M, Izadirad M, Bakhtiyaridovvombaygi M, Hasanpour SZ, Gharehbaghian A. Royal jelly induces ROS-mediated apoptosis in acute lymphoblastic leukemia (ALL)-derived Nalm-6 cells: shedding light on novel therapeutic approaches for ALL. Iran J Basic Med Sci. 2024;27:801–12. 10.22038/IJBMS.2024.76261.16498. 38800032 10.22038/IJBMS.2024.76261.16498 PMC11127081

[29] Abdelnour SA, Abd El-Hack ME, Alagawany M, Taha AE, Elnesr SS, Abd Elmonem OM, Swelum AA. Useful impacts of royal jelly on reproductive sides, fertility rate and sperm traits of animals. J Anim Physiol Anim Nutr (Berl). 2020;104:1798–808. 10.1111/jpn.13303. 31916638 10.1111/jpn.13303

[30] Kridli RT, Husein MQ, Humphrey WD. Effect of royal jelly and GnRH on the estrus synchronization and pregnancy rate in ewes using intravaginal sponges. Small Rumin Res. 2003;49:25–30. 10.1016/S0921-4488(03)00057-9.

[31] Zahmatkesh E, Najafi G, Nejati V, Heidari R. Protective effect of royal jelly on the sperm parameters and testosterone level and lipid peroxidation in adult mice treated with oxymetholone. Avicenna J Phytomed. 2014;4:43–52. 25050300 PMC4103725

[32] Alonso-Alconada L, deLa-Fuente A, Santacana M, Ferreiros A, Lopez-Lopez R, Matias-Guiu X, Abal M. Biomimetic device and foreign body reaction cooperate for efficient tumour cell capture in murine advanced ovarian cancer. Dis Model Mech. 2020;13:36–53. 10.1242/dmm.043653. 10.1242/dmm.043653 PMC7328160 32764154

[33] Kaçar AK, Ersöz M, Sezekler I, Coşkun ZM. The Effects of *Camellia sinensis* extract on proliferation, apoptosis and oxidative stress in insulinoma INS-1 cells. Bezmialem Sci. 2019;7:80–5. 10.14235/bas.galenos.2018.2287.

[34] Nakamura H, Wang Y, Kurita T, Adomat H, Cunha GR, Wang Y. Genistein increases epidermal growth factor receptor signaling and promotes tumor progression in advanced human prostate cancer. PLoS ONE. 2011;6:e20034. 10.1371/journal.pone.0020034. 21603581 10.1371/journal.pone.0020034 PMC3095647

[35] Jain A, Maheshwari V, Alam K, Mehdi G, Sharma SC. Apoptosis in premalignant and malignant squamous cell lesions of the oral cavity: a light microscopic study. Indian J Pathol Microbiol. 2009;52:164–6. 10.4103/0377-4929.48907. 19332902 10.4103/0377-4929.48907

[36] Moskwa J, Naliwajko SK, Dobiecka D, Socha K. Bee products and colorectal cancer-active components and mechanism of action. Nutrients. 2023;15:1614. 10.3390/nu15071614. 37049455 10.3390/nu15071614 PMC10097172

[37] Oršolić N, Jazvinšćak JM. Royal Jelly: Biological action and health benefits. Int J Mol Sci. 2024;25:6023. 10.3390/ijms25116023. 38892209 10.3390/ijms25116023 PMC11172503

[38] Okic-Djordjevic I, Trivanovic D, Krstic J, Jaukovic A, Mojsilovic S, Santibanez JF. GE132+Natural: Novel promising dietetic supplement with antiproliferative influence on prostate, colon, and breastcancer cells. J BUON. 2013;18:504–10. 23818369

[39] Peng CC, Sun HT, Lin IP, Kuo PC, Li JC. The functional property of royal jelly 10-hydroxy-2-decenoic acid as a melanogenesis inhibitor. BMC Complement Altern Med. 2017;17:392. 10.1186/s12906-017-1888-8. 28793915 10.1186/s12906-017-1888-8 PMC5550932

[40] Mustafa M, Ahmad R, Tantry IQ, Ahmad W, Siddiqui S, Alam M, Abbas K, Moinuddin, Hassan MI, Habib S, Islam S. Apoptosis: A comprehensive overview of signaling pathways, morphological changes, and physiological significance and therapeutic implications. Cells. 2024;13:1838. 10.3390/cells13221838. 39594587 10.3390/cells13221838 PMC11592877

[41] Green DR. Caspases and their substrates. Cold Spring Harb Perspect Biol. 2022;14:a041012. 10.1101/cshperspect.a041012. 35232877 10.1101/cshperspect.a041012 PMC8886984

[42] Ayna A, Tunç A, Özbolat S, Bengü AŞ, Aykutoğlu G. Anticancer, and antioxidant activities of Royal Jelly on HT-29 colon cancer cells and melissopalynological analysis. Turk J Bot. 2021;45:809–19. 10.3906/bot-2109-8.

[43] Albalawi AE, Althobaiti NA, Alrdahe SS, Alhasani RH, Alaryani FS, BinMowyna MN. Antitumor activity of royal jelly and its cellular mechanisms against Ehrlich Solid Tumor in mice. Biomed Res Int. 2022;11:7233997. 10.1155/2022/7233997. 10.1155/2022/7233997 PMC9071879 35528154

[44] Kocot J, Kiełczykowska M, Luchowska-Kocot D, Kurzepa J, Musik I. Antioxidant potential of propolis, bee pollen, and royal jelly: possible medical application. Oxid Med Cell Longev. 2018;18:1–29. 10.1155/2018/7074209. 10.1155/2018/7074209 PMC5954854 29854089

[45] Zhang S, Shao Q, Geng H, Su S. The effect of royal jelly on the growth of breast cancer in mice. Oncol Lett. 2017;14:7615–21. 10.3892/ol.2017.7078. 29344209 10.3892/ol.2017.7078 PMC5755229

[46] Wang CW, Chen CL, Wang CK, Chang YJ, Jian JY, Lin CS, Tai CJ, Tai CJ. Cisplatin-, Doxorubicin-, and Docetaxel-Induced cell death promoted by the aqueous extract of *Solanum* nigrum in human ovarian carcinoma cells. Integr Cancer Ther. 2015;14:546–55. 10.1177/1534735415588826. 26069278 10.1177/1534735415588826

